# Genetic alterations in a primary medullary thyroid carcinoma and its lymph node metastasis in a patient with 15 years follow-up

**DOI:** 10.1186/1746-1596-7-63

**Published:** 2012-06-07

**Authors:** Beatriz González-Yebra, Raúl Peralta, Ana Lilia González, Marco Antonio Ayala-Garcia, María E Medrano Ortiz de Zarate, Mauricio Salcedo

**Affiliations:** 1 Departamento de Medicina y Nutrición, División de Ciencias de la Salud, Campus León, Universidad de Guanajuato, Guanajuato, Mexico; 2 Departamento de Ciencias Aplicadas al Trabajo, División de Ciencias de la Salud, Campus León, Universidad de Guanajuato, Guanajuato, Mexico; 3 Hospital Regional de Alta Especialidad del Bajío, León, Guanajuato, México; 4 Departamento de Endocrinología, Hospital de Oncología, Centro Médico Nacional Siglo XXI-IMSS, México, DF, México; 5 Laboratorio de Oncología Genómica, Unidad de Investigación Médica en Enfermedades Oncológicas, Hospital de Oncología, CMN SXXI-IMSS, Av. Cuauhtémoc 330, Col. Doctores, México, DF 06720, Mexico

**Keywords:** Sporadic MTC, M918T *RET* mutation, Chromosomal alterations

## Abstract

**Background:**

Association between DNA alterations and clinical parameters as recurrence, survival or prognosis has been found in a variety of tumors. A clear association between Medullary Thyroid Carcinoma (MTC) and RET oncogene mutation has been accepted. Specifically M918T RET mutation represents the main genetic event in most cases of sporadic MTC (SMTC) and limited chromosomal alterations analyses have been performed.

**Methods:**

In the present work, a comparative genomic hybridization (CGH) study was performed using DNA from a primary tumor in a M918T RET mutation-positive SMTC patient and from its lymph node metastasis to investigate additional genetic alterations. We studied a patient with 15 years of follow-up and persistence of disease, confirmed by periodical elevated serum calcitonin (CT) levels.

**Results:**

Only 3 chromosomal imbalances were identified in the primary tumor, gain of 18p, and loss of 6p and 16p region, whereas 25 chromosomal imbalances were identified in the metastasis (9 gains and 16 losses).

**Conclusion:**

The chromosomal changes 6p-, 16p-, 18p + could determine in part the oncogenic phenotype in the primary M918T RET positive tumor and probably related to persistence of high serum CT levels in this patient. The additional chromosomal changes observed could be related to the metastasis phenotype. We suggest that some genes mapped at 6p, 16p and 18p chromosomal regions, could act as genes associated to cancer and could be related to persistent SMTC and good prognosis.

**Virtual slides:**

The virtual slide(s) for this article can be found here: http://www.diagnosticpathology.diagnomx.eu/vs/1720753793691097

## Background

Medullary thyroid carcinoma (MTC) is a neuroendocrine tumor arising from the parafollicular cells of the thyroid gland and approximately 75% of cases are sporadic type (SMTC) [1]. Survival rate is closely related to clinical parameters such as tumor stage, age of diagnosis, elevated postoperative serum calcitonin (CT) levels, and recurrence of the tumor [2]. Other parameters that contribute to survival rates include lymph node metastasis, tumor size >4 cm, extrathyroid and extranodal tumor extensions [3]. Thus, patients who are treated in the earlier stages of the disease have a better prognosis. Patients with tumors that are limited to the thyroid (stage I) have a survival rate of 95% at 10 and 20 years. Patients with advanced tumors, stages III and IV, have a survival rate of 55% and 28% at 10 and 20 years, respectively. Frequently, persistence of the disease is associated with elevated postoperative serum CT levels and persistence of the tumor increased over time. However, 30% of SMTC patients continued to show only elevated CT levels without additional clinical evidence of the disease (persistence), even after 15 years [2,3].

At genetic level, the first event in SMTC tumorigenesis is thought to be the M918T *RET* mutation (ATG → ACG) [4-11]. This mutation is associated with 30–66% of SMTC and 95% of Multiple Endocrine Neoplasia type 2B [4-9]. This mutation has been associated with a poor prognosis, tumor recurrence and the presence of lymph node metastases [10-13].

Comparative genomic hybridization (CGH) studies have been widely applied to cancer research [14,15]. CGH analysis has been performed in SMTCs showing common chromosomal alterations as loss in 1p, 3q, 4q, 13q, 17, 22q and gains in 11q, 12p, 14q, 19q and 22q (Table 1). Some of these imbalances have been related to specific clinical behavior such as the clinical outcome [16].

**Table 1:** Chromosomal alterations reported in carcinoma tissues from SMTC using CGH analysis

**Tissue**	**n**	**Ret mut**	**Gains**	**Losses**	**Reference**
SMTC primary tumor	10	Unknown	1q, 6, 7p, 11q, 21q	1p, 3q, 3, **13**, 18p, 18q, **22**	Hemmer et al., 1999 [18]
SMTC primary tumor	9	M918T	1p, 5p, 7q, 8q, 9q, **11q**, 16, 19p, **19q**, **22q**	**1p**, 2q, 3q, 3, 4q, 9p + q, 10, 12p, 12q, **13q**.	Frisk et al., 2001 [16]
SMTC primary tumor	12	M918T	6p, 6, 7, **12p**, **14q,** 15q, 16, **19**, X	**1p**, 3q, **3**, **4q**, 5q, 9, 11, **13q**, 16p,16, 17p, **17**, 19, 20q, 20, **22q**, **X**	Marsh et al., 2003 [19]
SMTC primary tumor	1	M918T	18p	6p , 16p	Present case
SMTC metastasis	1	M918T	6 ,14q	X	Marsh et al., 2003 [19]
SMTC metastasis	1	M918T	2q, 3q, 4p + q, 5p + q, 6q, 8q, 12p + q, 13q, Xq	1p, 1q, 6p, 8p, 8q, 9q, 10p, 10q, 11q, 12q, 15q, 16, 17, 19, 20, and 22q	Present case

Note: n: number of cases studied; Ret mut: type of RET mutation; M918T: codon 918 (Met– > Thr). In bold letter the most common CGH gains and losses. Bold font indicates most common CGH imbalances reported by author.

In order to know which chromosomal imbalances occur in a primary SMTC harboring a M918T RET mutation and its lymph node metastasis in a patient with 15 years of clinical follow-up, a CGH analysis was performed.

## Case presentation

In this study, we report a case of a 70 year-old female who was diagnosed with SMTC 15 years ago. A total resection of the thyroid gland and a resection of the right lymph nodes in the neck were performed at age 55. At the time of surgery, a unilateral tumor was detected in the right lobe of the thyroid. Histopathological findings confirmed a 7.5x3.5x2cm tumor (T2) with extraglandular extension, 8/17 positive lymph nodes metastases (N1b) and no distant metastases (M0) were found. According to TNM classification, this patient was diagnosed as MTC stage III. One year later, the patient presented with persistently high serum CT levels (2,400 pg/mL). During a second surgery, a left lymph nodes resection was performed and histopathological analysis confirmed the presence of several lymph node metastases (10/49). At present, the patient is alive and has persistent disease, confirmed by periodical elevated serum CT levels (3,193 pg/mL at the latest screening).

Molecular diagnostic testing confirmed the presence of the M918T RET mutation in the tumor and lymph node metastases previously. Interestingly, our case study demonstrate that despite the mutation and the presence of several lymph nodes metastases, the patient experienced a good survival rate (15 years after surgery) and still alive. Then, we focused in search of genetic DNA alterations by CGH that could be important in the favorable prognosis of this patient. The CGH procedure was performed using reagents and kits from Vysis Inc. (Downers Grove, IL), following the manufacturer’s instructions. Tumoral DNA was labeled using the CGH nick translation kit and the hybridization mixture was prepared according to the CGH reagent kit, consisting of 200 ng of spectrum-green labeled tumoral DNA, 200 ng of spectrum-red labeled normal male reference DNA and 20 μg of human Cot-1 DNA. Hybridization was performed over 3 days at 37 °C on normal male metaphase spreads (Vysis, Downers Grove, IL). Digital images were collected using the Smart Capture (Vysis) software with a charged coupled device camera mounted on an epifluorescence microscope. In the primary tumor, the chromosomal alterations detected were losses in 6p and 16p and gain in 18p, while 25 chromosomal alterations were detected in the lymph node metastasis with more losses than gains (Table 1).

## Discussion

**Figure 1:**
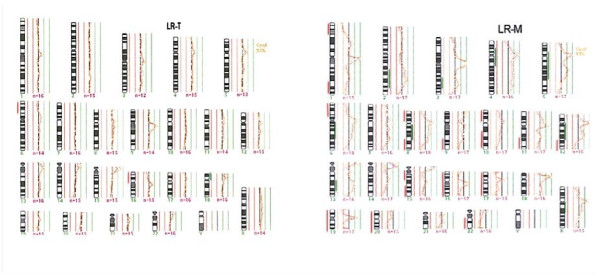
**CGH analysis profile from SMTC tissues: A) Primary tumor, B) Metastasis.** Red lines to the left of chromosomes represent losses (CGH fluorescence ratios <0.80), green lines to the right of chromosomes represent gains (ratio >1.20). Abbreviations: LR-T: Primary tumor, LR-M: Metastasis.

Cancer progression and the metastasis result from the accumulation of multiple genetic changes. In the current study, DNA from a primary tumor and a lymph node metastasis of a persistent SMTC patient, harboring the M918T RET mutation, was subjected to CGH analysis to evaluate additional genetic changes. This analysis demonstrated differences in genetic changes between the primary tumor and the metastasis, 3 genetic changes vs. 25 genetic changes, respectively. Interestingly, DNA losses were more common than gains, and a complete deletion of chromosomes 16, 17, 19, and 20 were observed in the metastasis (Figure 1). In contrast, a high number of chromosomal alterations in lymph nodes metastasis have been previously found [16]. These findings could indicate a complex pattern of DNA alterations related to metastasis in this patient. It is important to note that the chromosomal alterations observed in the primary tumor are also present in its metastasis, indicating that those changes could be associated to clonal origin. This is also supported by the presence of the M918T RET mutation in the metastasis.

To date, only few studies have analyzed chromosomal imbalances in primary tissue of SMTC [16-18]. Our data support previous results indicating that the SMTC could be characterized by more DNA losses than gains [16,18]. One report had suggested that the SMTC harboring the M918T RET mutation have a higher degree of chromosomal instability and usually display a higher number of DNA alterations [16]. However, the fewer number of DNA alterations present in the primary tumor of our patient could be related to persistence or good prognosis. If this is true, the low number of specific DNA alterations in the primary tumor could be of prognostic value in SMTC patients.

In other study, the loss of chromosomes 1p, 3q 13q and 22q were detected in some SMTC patients with M918T RET mutation. However, the chromosomal alterations in the metastasis described in the present study, are partially similar than those described for the primary tumor in other reports (Table 1). Therefore, it is possible that some of these DNA alterations could be associated with clinical parameters such as prognosis, tumor size, and metastasis. For instance, gains of chromosome 19 and chromosome 11q12 were observed in patients whom died of the disease, and they also harbored the M918T mutation [16]. It is clear that heterogeneity in chromosomal alterations is present in SMTCs. Several authors have suggested that these CGH imbalances could contribute to outcome or prognosis and that those genetic regions may contain genes associated to the development of SMTC [16-19]. An overexpression of genes involved in proliferation and invasion such as PTN (7q33); ESM1 (5q11), and CEACAM (19q13) could also characterize an aggressive SMTC [20]. Thus, our results are supported by these previous findings indicating that chromosomal changes at 6p, 16p and 18p observed in the SMTC, could not be associated with an aggressive prognosis. On the other hand, it is widely known that the M918T RET mutation is associated with poor prognosis and persistence of the disease [9-12]. According to our results, the patient harboring the M918T RET mutation failed in the poor prognosis, however, she had persistence SMTC. In this case we hypothesized that our patient harboring the RET mutation (aggressive mutation) could be subjected to an epigenetic mechanism as methylation (for instance, an imprinted mutated allele) allowing the long survival (good prognosis). Based on our results, we suggest that: 6p, 16p, and 18p regions could contain genes associated to cancer that are common in human cancer genome [21,22] and the loss or gain could be associated to good prognosis and long-term survival. More detailed analyses are needed to support the existence of good prognostic markers in chromosomal regions that contain imbalances. To avoid a limited significance about genetic alterations in persistence SMTC harboring M918T RET mutation patients should be necessary to analyze more of these kind of patients.

## Conclusion

In summary, the chromosomal alterations in SMTC are quite heterogeneous; however, the number and specific DNA alterations could mark our patient’s prognosis even when harbors the M918T RET mutation. Losses in 6p and 16p, and gain in 18p could be related to a favorable prognostic outcome, persist disease and high serum CT levels in SMTC.

## Consent

Written informed consent was obtained from the patient for publication of this case report and any accompanying images. A copy of the written consent is available for review by the Editor-in-Chief of this Journal.

## Competing interest

The authors declare that they have no competing interests.

## Authors’ contributions

BG and MS conceived the study, participated in its design and coordination and helped to draft the manuscript. RP, MA, MM and ALG carried out the molecular studies and drafted the manuscript. All authors read and approved the final manuscript.

## References

[B1] Randolph GW Maniar D Medullary carcinoma of the thyroid Cancer Control 2000 7 253 261 10832112 10.1177/107327480000700305

[B2] Girelli ME Nacamulli D Pelizzo MR De Vido D Mian C Piccolo M Busnardo B Medullary thyroid carcinoma: clinical features and long-term follow-up of seventy-eight patients treated between 1969 and 1986 Thyroid 1998 8 517 523 10.1089/thy.1998.8.517 9669290

[B3] Ito Y Miyauchi A Yabuta T Fukushima M Inoue H Tomoda C Uruno T Kihara M Higashiyama T Takamura Y Miya A Kobayashi K Matsuzuka F Alternative surgical strategies and favorable outcomes in patients with medullary thyroid carcinoma in Japan: experience of a single institution World J Surg 2009 33 1 58 66 10.1007/s00268-008-9795-2 19005720

[B4] González B Salcedo M Medrano ME Mantilla A Quiñonez G Benítez-Bibriesca L Rodríguez-Cuevas S Cabrera L de León B Altamirano N Tapia J Dawson B RET oncogene mutations in medullary thyroid carcinoma in Mexican families Arch Med Res 2003 34 1 41 49 10.1016/S0188-4409(02)00461-7 12604374

[B5] Hofstra RM Landsvater RM Ceccherini I Stulp RP Stelwagen T Luo Y Pasini B Höppener JW van Amstel HK Romeo G A mutation in the RET proto-oncogene associated with multiple endocrine neoplasia type 2B and sporadic medullary thyroid carcinoma Nature 1994 367 375 376 10.1038/367375a0 7906866

[B6] Eng C Smith DP Mulligan LM Nagai MA Healey CS Ponder MA Gardner E Scheumann GF Jackson CE Tunnacliffe A Point mutation within the tyrosine kinase domain of the RET proto-oncogene in multiple endocrine neoplasia type 2B and related sporadic tumors Hum Mol Genet 1994 3 237 241 10.1093/hmg/3.2.237 7911697

[B7] Zedenius J Wallin G Hamberger B Nordenskjöld M Weber G Larsson C Somatic and MEN2A de novo mutations identified in the RET proto-oncogene by screening of sporadic MTC:s Hum Mol Genet 1994 3 1259 1262 10.1093/hmg/3.8.1259 7987299

[B8] Eng C Mulligan LM Smith DP Healey CS Frilling A Raue F Neumann HP Pfragner R Behmel A Lorenzo MJ Mutation of the RET protooncogene in sporadic medullary thyroid carcinoma Genes Chromosomes Cancer 1995 12 209 212 10.1002/gcc.2870120308 7536460

[B9] Scurinni C Quadro L Fattoruso O Verga U Libroia A Lupoli G Cascone E Marzano L Paracchi S Busnardo B Girelli ME Bellastella A Colantuoni V Germline and somatic mutations of the RET proto-oncogene in apparently sporadic medullary thyroid carcinomas Mol Cell Endocrinol 1998 137 51 57 10.1016/S0303-7207(97)00234-7 9607728

[B10] Zedenius J Larsson C Bergholm U Bovée J Svensson A Hallengren B Grimelius L Bäckdahl M Weber G Wallin G Mutations of codon 918 in the RET proto-oncogene correlate to poor prognosis in sporadic medullary thyroid carcinomas J Clin Endocrinol Metabol 1995 80 3088 3090 10.1210/jc.80.10.3088 7559902

[B11] Schilling T Bürk J Sinn HP Clemens A Otto HF Höppner W Herfarth C Ziegler R Schwab M Raue F Prognostic value of codon 918 (ATG → ACG) RET proto-oncogene mutations in sporadic medullary thyroid carcinoma Int J Cancer 2001 95 62 66 10.1002/1097-0215(20010120)95:1<62::AID-IJC1011>3.0.CO;2-1 11241313

[B12] Romei C Elisei R Pinchera A Ceccherini I Molinaro E Mancusi F Martino E Romeo G Pacini F Somatic mutations of the RET protooncogene in sporadic medullary thyroid carcinoma are not restricted to exon 16 and are associated with tumor recurrence J Clin Endocrinol Metabol 1996 81 1619 1622 10.1210/jc.81.4.1619 8636377

[B13] Elisei R Cosci B Romei C Bottici V Renzini G Molinaro E Agate L Vivaldi A Faviana P Basolo F Miccoli P Berti P Pacini F Pinchera A Prognostic significance of somatic RET oncogene mutations in sporadic medullary thyroid cancer: a 10-year follow-up study J Clin Endocrinol Metabol 2008 93 682 687 10.1210/jc.2007-1714 18073307

[B14] Pinkel D Albertson DG Comparative genomic hybridization Annu Rev Genomics Hum Genet 2005 6 331 354 10.1146/annurev.genom.6.080604.162140 16124865

[B15] Weiss MM Hermsen MA Meijer GA van Grieken NC Baak JP Kuipers EJ van Diest PJ Comparative genomic hybridization Mol Pathol 1999 52 243 251 10.1136/mp.52.5.243 10748872 PMC395705

[B16] Frisk T Zedenius J Lundberg J Wallin G Kytölä S Larsson C CGH alterations in medullary thyroid carcinomas in relation to the RET M918T mutation and clinical outcome Int J Oncol 2001 18 1219 1225 11351254 10.3892/ijo.18.6.1219

[B17] Ye L Santarpia L Cote GJ El-Naggar AK Gagel RF High resolution array-comparative genomic hybridization profiling reveals deoxyribonucleic acid copy number alterations associated with medullary thyroid carcinoma J Clin Endocrinol Metab 2008 93 4367 4372 10.1210/jc.2008-0912 18765511 PMC2729231

[B18] Hemmer S Wasenius VM Knuutila S Franssila K Joensuu H DNA copy number changes in thyroid carcinoma Am J Surg Pathol 1999 154 1539 1547 10.1016/S0002-9440(10)65407-7 PMC1866579 10329606

[B19] Marsh DJ Theodosopoulos G Martin-Schulte K Richardson AL Philips J Röher HD Delbridge L Robinson BG Genome-wide copy number imbalances identified in familial and sporadic medullary thyroid carcinoma J Clin Endocrinol Metab 2003 88 1866 1872 10.1210/jc.2002-021155 12679485

[B20] Ameur N Lacroix L Roucan S Roux V Broutin S Talbot M Dupuy C Caillou B Schlumberger M Bidart JM Aggressive inherited and sporadic medullary thyroid carcinomas display similar oncogenic pathways Endocr Relat Cancer 2009 16 1261 1272 10.1677/ERC-08-0289 19675075

[B21] Santarius T Shipley J Brewer D Stratton MR Cooper CS A census of amplified and overexpressed human cancer genes Nat Rev Cancer 2010 10 59 64 10.1038/nrc2771 20029424

[B22] Knuutila S Björkqvist AM Autio K Tarkkanen M Wolf M Monni O Szymanska J Larramendy ML Tapper J Pere H El-Rifai W Hemmer S Wasenius VM Vidgren V Zhu Y DNA copy number amplification in human neoplasm Am J Pathol 1998 152 1107 1123 9588877 PMC1858578

